# Brief Report: Best Discriminators for Identifying Children with Autism Spectrum Disorder at an 18-Month Health Check-Up in Japan

**DOI:** 10.1007/s10803-015-2527-1

**Published:** 2015-07-19

**Authors:** Yoko Kamio, Hideyuki Haraguchi, Andrew Stickley, Kazuo Ogino, Makoto Ishitobi, Hidetoshi Takahashi

**Affiliations:** Department of Child and Adolescent Mental Health, National Institute of Mental Health, National Center of Neurology and Psychiatry, 4-1-1 Ogawahigashi-cho, Kodaira-shi, Tokyo, 187-8553 Japan; Department of Human Ecology, Graduate School of Medicine, University of Tokyo, Tokyo, Japan; Stockholm Centre for Health and Social Change (SCOHOST), Södertörn University, Huddinge, Sweden; Department of Child and Adolescent Psychiatry, Tokyo Metropolitan Children’s Medical Center, Fuchu-shi, Tokyo, Japan

**Keywords:** Autism spectrum disorder, Screening, Short form, Modified checklist for autism in toddlers (M-CHAT), Primary care settings

## Abstract

To determine the best discriminative items for identifying young children with autism spectrum disorders (ASD), we conducted a secondary analysis using longitudinal cohort data that included the Japanese version of the 23-item modified checklist for autism in toddlers (M-CHAT-JV). M-CHAT-JV data at 18 months of age and diagnostic information evaluated at age 3 or later from 1851 Japanese children was used to isolate six highly discriminative items. Using data from two different community samples (n = 1851, n = 665) these items were shown to have comparable psychometric values with those of the full version. Our results suggest that these items might work as a short form screener for early identification of ASD in primary care settings where there are time constraints on screening.

## Introduction

A growing body of research data supports the idea that community-based active surveillance for early signs of autism spectrum disorders (ASDs), complemented by autism-specific screening tools, may help reduce the time between the initial emergence of ASD symptoms and referral for specialized assessment (Robins 2008; Zwaigenbaum et al. 2009). As recommended by the American Academy of Pediatrics (2006), screening at 18 and again at 24 months regardless of whether any concern has been raised during the surveillance process is likely to identify the largest number of children without compromising specificity (Barton et al. 2012).

The modified checklist for autism in toddlers (M-CHAT) (Robins et al. 2001) is a 23-item parent-report instrument for children aged 16–30 months. It is one element in a “two-step” screening procedure that consists of the initial administration of the M-CHAT screening questionnaire (step 1) and the M-CHAT Follow-Up Interview (M-CHAT/F) (www.mchatscreen.com/Official_M-CHAT_Website_files/M-CHAT_FollowUp.pdf) (step 2). The M-CHAT/F, which has been recommended based on the findings from studies targeting low-risk children (Chlebowski et al. 2013; Kleinman et al. 2008; Pandey et al. 2008; Robins 2008), confirms whether positive screens still fail on certain items, resulting in a reduced number of false positive (FP) cases and therefore improving the positive predictive value (PPV). A recent large-scale study reported that M-CHAT screening for children aged 18–24 months in primary care settings resulted in a PPV of 0.54 for identifying ASD, and 0.98 for identifying any developmental concerns (Chlebowski et al. 2013).

In Japan, 18-month-old children have been eligible to receive free health check-ups at local public health centers or pediatric clinics since 1965 by law. Since the main focus is general developmental screening for motor, cognitive, or language developmental problems, adding autism-specific screening to the routine check-up is expected to facilitate the earlier referral of children for further services who will be later diagnosed with ASD, which would heighten the value of community-based developmental surveillance (Zwaigenbaum et al. 2009). To examine the usefulness of M-CHAT for identifying Japanese toddlers with ASD at the 18-month routine health check-up, in an earlier study, a total of 1851 children were screened with the Japanese version of this instrument (M-CHAT-JV) (Kamio et al. 2014). Final ASD diagnoses were confirmed at age ≥3 years via community-based surveillance. This two-step screening procedure resulted in a sensitivity of 0.48, a specificity of 0.99, a PPV of 0.46, and a likelihood ratio (LR) of 33.4. The PPV was similar to that seen in the Chlebowski et al. (2013) study, but even higher than that reported by Pandey et al. (2008), despite the fact that the children were younger (18 months rather than 24 months of age) and that a slightly modified threshold was used (Kamio et al. 2014).

Besides diagnostic accuracy, a notable aspect of the above-described process was the high rate of attrition and/or follow-up refusal, which has been consistently observed in ASD screening of younger/low-risk children (Dietz et al. 2006; Khowaja et al. 2015; Pandey et al. 2008), and might lower PPVs. In relation to this, Pandey et al. (2008) suggested that parents of low-risk 18-month-old children might need more support through a two-step screening process compared to those of low-risk 24-month-old children, while Robins (2008) stated that the use of the follow-up interview for screen-positive children on the M-CHAT questionnaire should be integrated into the well-child visit, which helps determine the need for referral immediately.

Considering the tight time constraints in primary care settings, a brief screening tool might be helpful in facilitating the integration of autism-specific screening within routine general developmental screening. Specifically, it would be easier for professionals with minimal experience of ASD to have an instrument with a limited number of items both in terms of its administration as well as when it comes to explaining to parents about the significance of the test for early development. Moreover, it may elicit greater parental cooperation and permit direct discussion of potential concerns (Barton et al. 2012).

Given this, the goal of this exploratory study was to identify the best discriminators of ASD among the 23 items of the M-CHAT-JV, and examine the reliability, validity, and diagnostic accuracy of the selected item set using cohort data from 2 geographic regions.

## Methods

### Participants

Data from two prospective community cohorts in Japan, Fukuoka (cohort 1) and Tokyo (cohort 2) were included in a secondary analysis. Cohort 1 comprised 1851 children (942 boys) who received health check-ups when aged 18 months between April 2004 and March 2007 and again at 3 years of age at a local health center. From these, 51 were confirmed as having ASD when aged 3 or above (ASD-1 group), while 1800 children were judged not to have ASD at age 5 via developmental surveillance in the community (community control-1 group). The details of the data collection have been described elsewhere previously (Kamio et al. 2014). These 1851 children represented 82 % of the total child population of that age group that resided in Fukuoka during the study period. Cohort 2 comprised 665 children (342 boys) who received health check-ups at age 18 months between November 2008 and October 2009 and at 2 years of age at a local health center in Tokyo. Thirteen of these children were confirmed as having ASD when aged 2 or above (ASD-2 group), while 652 children were classified as belonging to community control-2 group. The details of the data collection are described in a previous report (Koyama et al. 2010). These 665 children represented 87 % of all the children who attended the health check-up at a local health center during the specified time period.

Fourteen children in cohort 1 and 3 children in cohort 2 receiving medical treatment or therapeutic interventions because of severe cognitive or motor developmental delays at 18 months were excluded from this study, in light of the additional load this might place on the parents. The ASD diagnosis was confirmed by our research team according to DSM-IV-TR criteria based on a comprehensive evaluation that included the Japanese version of the Autism Diagnostic Interview-Revised (ADI-R) (Tsuchiya et al. 2013), the Autism Diagnostic Observation Schedule (ADOS) (Kuroda et al. 2013), and the Childhood Autism Rating Scale (CARS) (Kurita et al. 1989). IQs/DQs in cohort 1 were assessed using different measures depending on mental age, such as the Tanaka-Binet Intelligence Scale V for children, Enjoji’s Analytical Developmental Test for children under age 4, or the Japanese version of the Wechsler Intelligence Scale for Children-Third Edition (WISC-III) at age 5. DQs in cohort 2 were assessed using the Kyoto Scale of Psychological Development Test (KSPD), which is widely used in Japanese clinical settings for young and/or developmentally delayed children, and has been shown to be comparable to the Bayley Scales of Infant Development second edition (Tatsuta et al. 2013). The KSPD DQs have been found to be comparable to IQ scores for children with pervasive developmental disorders (PDD) (Koyama et al. 2009; Table 1).

**Table 1:** Characteristics of the study participants

	Cohort 1 (*n* = 1851)		Cohort 2 (*n* = 665)	
	ASD-1 (*n* = 51) mean (*SD*), range	Community control-1 (*n* = 1800) mean (*SD*), range	ASD-2 (*n* = 13) mean (*SD*), range	Community control-2 (*n* = 652) mean (*SD*), range
Gender, male:female	35:16	907:893	8:5	334:318
Age at M-CHAT-JV (months)	18.6 (0.6) 18–21	18.7 (0.6) 17–26	19.2 (0.7) 18–20	18.7 (0.7) 17–23
Age at final diagnostic evaluation (months)	50.6 (14.2) 33–73	–	37.6 (10.8) 24–53	–
IQ/DQ	80.1 (26.7) 20–134^a^	–	75.6 (10.9) 55–99	–
≥70	25 (49.0 %)		10 (76.9 %)	
<70	26 (51.0 %)		3 (23.1 %)	
ADI-R total	25.5 (7.5) 11–39^b^	–	29.9 (6.7) 19–41	–
ADOS (a) + (b) total	13.4 (3.8) 9–23^c^	–	14.9 (3.2) 10–20^d^	–
CARS	34.0 (4.7) 24.5–44.5	–	34.8 (4.0) 25–41	–

*M*-*CHAT*-*JV* modified checklist for autism in toddlers-Japanese version

^a^Forty-three out of 51 children were evaluated by standardized cognitive tests

^b^Thirty out of 51 children were evaluated using the ADI-R

^c^Nineteen out of 51 children were evaluated using the ADOS

^d^Twelve out of 13 children were evaluated using the ADOS

### Measurements

The 23-item M-CHAT-JV was culturally and linguistically adapted with the authorization of the original authors. Previous research has shown that the M-CHAT-JV score (the number of failed items) was significantly correlated among mother-father pairs, representing good inter-rater reliability. The test–retest reliability was good; mothers provided near identical M-CHAT-JV scores on two different occasions. In addition, the significant correlation of total scores with the CARS scores indicates sufficient concurrent validity. Moreover, the total scores were significantly different among children with autism, PDD not otherwise specified (PDD-NOS), and control children in an expected direction suggesting a high degree of discriminant validity (Inada et al. 2011). Based on prospective data from cohort 1, the use of the M-CHAT-JV for 18-month old low-risk children was shown to result in a PPV of 0.12, while its use with a follow-up interview increased the PPV to 0.46 (Kamio et al. 2014).

Before the health check-up, questionnaires were sent to the mothers to obtain information about the family structure, maternal mental health, and the child’s feeding, sleep, play, illness, development and behavior. The M-CHAT-JV items were included in the section on the child’s behavior. Mothers of cohort 1 children responded to the 23 M-CHAT-JV items about their 18-month-old children, from which the most highly discriminating items were subsequently selected. Mothers of cohort 2 children completed selected M-CHAT-JV items about their children when aged 18 months, and the full 23 items at age 2.

### Statistical Analyses

Item selection was based on cohort 1 data. First, we compared the failure rate for each of the 23 items between the ASD-1 and community control-1 groups using Fisher’s exact test with a Bonferroni correction. Next, a discriminant function analysis was conducted to identify the best set of items that predicted group membership. To examine the internal consistency of the selected item set, Cronbach’s α was calculated for all members of cohorts 1 and 2 and the ASD-1, ASD-2 groups. For its test–retest reliability, Pearson’s correlation coefficients were calculated for the M-CHAT scores (i.e., the number of failed items) for the cohort 2 sample. To examine predictive validity, we computed Pearson’s correlation coefficients between the selected item set scores at 18 months and clinical measurements assessed at 2 years of age for children in cohorts 1 and 2 who were fully evaluated because they were suspected of having ASD based on the M-CHAT-JV results, respectively. In addition, we conducted a receiver operating characteristics (ROC) analysis of the dataset from cohorts 1 and 2, respectively. The analysis was performed using SPSS 19.0 J for Windows.

### Ethical Considerations

This study was conducted with institutional review board approval. This study was performed in accordance with the ethical standards laid down in the 1964 Declaration of Helsinki and its later amendments. For this type of study formal consent is not required.

## Results

### Item Analysis of the 23 Items of the M-CHAT-JV

As shown in Table 2, the failure rate at 18 months of age was significantly higher in the ASD-1 group than in the community control-1 group for 16 items (*p*s < 0.05). Using a stringent *p* level (*p*s < 0.001), the following 11 items were selected: “pretend play”, “protoimperative pointing”, “protodeclarative pointing”, “functional play”, “brings objects to show”, “imitation of action”, “responds to name”, “point following”, “gaze following”, “language comprehension”, and “social referencing”.

**Table 2:** Failure rate for the 23 items of the M-CHAT-JV

Item	ASD-1 (*n* = 51)	Community control-1 (*n* = 1800)	*p* value
1: Enjoys being swung	0.02	0.06	0.054
2: Interest in other children	5.88	0.94	0.016*
3: Climbs up stairs	0.00	0.67	1.000
4: Enjoys peek-a-boo	1.96	0.22	0.131
5: Pretend play	33.33	3.78	0.000***
6: Protoimperative pointing	37.25	1.89	0.000***
7: Protodeclarative pointing	35.29	3.00	0.000***
8: Functional play	49.02	24.00	0.000***
9: Brings objects to show	29.41	3.72	0.000***
10: Eye contact	7.84	1.22	0.005**
11: Oversensitive to noise	13.73	15.73	0.846
12: Responds to smile	1.96	0.11	0.080
13: Imitation of action	31.37	1.61	0.000***
14: Responds to name	11.76	0.39	0.000***
15: Point following	23.53	1.39	0.000***
16: Walking	0.00	0.39	1.000
17: Gaze following	25.49	5.50	0.000***
18: Unusual finger movements	5.88	2.06	0.095
19: Gaining parent’s attention	13.73	3.33	0.002**
20: Auditory response	3.92	0.56	0.041*
21: Language comprehension	17.65	1.17	0.000***
22: Visual response	29.41	11.78	0.001**
23: Social referencing	33.33	10.28	0.000***

*M*-*CHAT*-*JV* modified checklist for autism in toddlers-Japanese version

### Discriminant Function Analysis of the 23 Items of the M-CHAT-JV

Table 3 shows standardized canonical discriminant function coefficients indicating the discriminant power of group membership, with the item order rearranged for the 11 items described above. Despite a high discriminant coefficient, item 14 was excluded as its kappa coefficients for inter-rater and test–retest agreement were reported as being non-significant in a previous study (−0.167, 0.327, respectively) (Inada et al. 2011). Six items with the highest standardized discriminant coefficients (“protoimperative pointing”, “imitation of action”, “pretend play”, “point following”, “language comprehension”, “brings objects to show”), were chosen as the best discriminators. The kappa coefficients of these 6 items for inter-rater and test–retest agreement were reported to range between 0.600–0.808 and 0.792–1.000, respectively (*p*s < 0.01) (Inada et al. 2011).

**Table 3:** Standardized canonical discriminant function coefficients for the M-CHAT-JV items that were significantly different between the ASD-1 and community control-1 groups

Item	Standardized discriminant coefficient
6: Protoimperative pointing	0.511
13: Imitation of action	0.437
14: Responds to name	0.335
5: Pretend play	0.197
15: Point following	0.153
21: Language comprehension	0.143
9: Brings objects to show	0.132
7: Protodeclarative pointing	−.100
8: Functional play	0.090
17: Gaze following	−.087
23: Social referencing	−.014

*M*-*CHAT*-*JV* modified checklist for autism in toddlers-Japanese version

### Internal Consistency of the 6-Item set of the M-CHAT-JV

Cronbach’s α for the 6-item set was 0.637 for the total cohort 1 sample, 0.751 for the ASD-1 group, 0.583 for the total cohort 2 sample, and 0.879 for the ASD-2 group. Alpha values were superior to those of the full version of the scale in cohort 1 (total cohort 0.590, ASD-1 0.741, respectively).

### Test–Retest Reliability of the 6-Item Set of the M-CHAT-JV

The 6-item scores at 18 months of age were significantly correlated with the 6-item scores at 2 years of age for the total sample in cohort 2 (*r* = 0.455, *p* < 0.01).

### Predictive Validity of the 6-Item Set of the M-CHAT-JV

In cohort 1, the 6-item scores at 18 months of age were significantly correlated with the CARS scores and DQ at age 2 in 38 children including 25 children diagnosed with ASD (*r* = 0.414, *p* = 0.01; −0.563, *p* < 0.01, respectively) and with the social and communication domain scores of the ADI-R in 22 children including 15 children diagnosed with ASD (*r* = 0.622, 0.585, *p*s < 0.01), while there were no correlations between the short form scores and the ADI-R repetitive behavior scores (*r* = −0.153).

In cohort 2, the 6-item scores at 18 months of age were significantly correlated with the full version scores at age 2 for the total sample (*r* = 0.513, *p* < 0.01). For 21 children who were fully evaluated including 13 children diagnosed with ASD, the 6-item scores were significantly correlated with the social domain scores of the ADI-R and DQs (*r* = 0.447, −0.452, *p*s < 0.05, respectively), but not with the CARS (*r* = 0.428, *p* = 0.05) or the communication domain scores of the ADI-R (*r* = 0.414, *p* = 0.06), which were marginally significant. The 6-item scores were not significantly correlated with either the ADI-R repetitive behavior scores or the ADOS scores (*r* = 0.148, 0.309, respectively).

The estimated area under the curve for the 6-item set was 0.800 [95 % confidence interval (CI) 0.720–0.879] and 0.749 (95 % CI 0.582–0.915) for cohorts 1 and 2, respectively, whereas that for the full version was 0.833 (95 % CI 0.760–0.906) for cohort 1 at 18 months of age. Table 4 shows the sensitivity, specificity, PPV, LR, and Youden’s index (sensitivity + specificity − 1) of the selected item set for predicting a later diagnosis of ASD at 18 months of age in cohorts 1 and 2 (cutoffs ≥1, 2, 3). Considering an optimal balance between sensitivity and specificity, failing 1 of the 6 items was chosen as the best cutoff for this age.

**Table 4:** ROC analysis results for the 6-item set of the M-CHAT-JV in cohort 1 and cohort 2 samples

Cutoff score	Sensitivity	95 % CI	Specificity	95 % CI	PPV	95 % CI	LR	95 % CI	Youden’s index
Cohort 1									
≥1	0.667	0.535–0.778	0.904	0.901–0.908	0.165	0.132–0.193	6.977	5.387–8.415	0.571
≥2	0.431	0.316–0.550	0.975	0.972–0.978	0.328	0.241–0.418	17.255	11.178–25.395	0.406
≥3	0.294	0.200–0.392	0.989	0.987–0.992	0.441	0.299–0.588	27.864	15.082–50.365	0.284
Cohort 2									
≥1	0.615	0.359–0.821	0.831	0.826–0.835	0.068	0.040–0.090	3.648	2.064–4.990	0.447
≥2	0.385	0.183–0.627	0.968	0.964–0.973	0.192	0.091–0.314	11.941	5.048–22.909	0.352
≥3	0.231	0.087–0.425	0.991	0.988–0.995	0.333	0.126–0.614	25.077	7.199–79.634	0.222

Values were calculated based on the total sample (ASD-1 51, community control-1 1851)

*CI* confidence interval, *PPV* positive predictive value, *LR* likelihood ratio

## Discussion

Using community-based cohort data obtained via long-term surveillance, this study identified a highly discriminative 6-item set from the 23-item M-CHAT-JV and demonstrated its reliability and validity with cohort data from 2 geographically different regions in Japan. The results of this secondary analysis suggest that as a first step in the screening of ASD at 18 months of age in primary care settings, the selected 6 items may have the potential to screen children with a similar PPV to the full version of M-CHAT-JV.

In this study, the items most discriminative for 18-month-old children were “protoimperative pointing”, “imitation of action”, “pretend play”, “point following”, “language comprehension”, and “brings objects to show”, i.e., 5 items related to preverbal social behaviors, while there was one language-related item. An earlier cross-sectional study in Japan (Inada et al. 2010) reported that the vast majority of parents in the community recognize “imitation of action”, “point following”, “pretend play”, and “protoimperative pointing” when their child is between 11 and 12 months old, and “brings objects to show” when the child is 15 months old, while almost all parents of children aged 18 months (92–100 %) observed these preverbal behaviors. In other words, consistent with the result of this study, the absence of any of these early social developmental manifestations in a child aged 18 months seems to be discernible to parents, even if parents do not explicitly address these concerns at this age.

When comparing our selected items with those from an earlier M-CHAT study in the United States that attempted to select a reduced number of items which it termed the Best7 (Robins et al. 2010), which were subsequently placed within the first 10 items in the new M-CHAT-R/F (Robins et al. 2014), 3 preverbal social items (“pretend play”, “brings objects to show”, and “point following”) emerge as common items which are most sensitive to ASD among children in the US and Japan. Moreover, when the present results are compared with those from M-CHAT studies undertaken earlier in Norway and China as well as with the findings from the US study mentioned above, it can be seen that in the four countries, “pretend play” and “brings objects to show” are common items that are the most sensitive to ASD at 18 months of age (Stenberg et al. 2014; Wong et al. 2004), and at 18–24 months of age (Robins et al. 2010). In addition, “point following” is an item that children with ASD commonly fail in the US, China and Japan, “imitation of action” is an item that children with ASD commonly fail in China and Japan, while the same thing occurs with “language comprehension” in Norway and Japan. Thus, our best items seem to be culturally universal.

The psychometric values when using a cutoff ≥1 out of 6 items were consistent with those of the full version (Kamio et al. 2014), and also with those of the original full version in 18–24 month old low-risk children in a recent U.S. study (PPV 0.05–0.06, Chlebowski et al. 2013). Indeed, the psychometric values previously reported for a preliminary 9-item short version of the M-CHAT-JV (sensitivity 0.650, specificity 0.885, PPV 0.088, Inada et al. 2011) almost remain unchanged for the current 6-item set, although the previous short version was only retrospectively tested on a smaller sample with a short follow-up period in just one area. On the other hand, the values for the Norwegian cohort aged 18 months were much lower (Stenberg et al. 2014). The differences between these studies might relate to variations in the whole procedure from screening to diagnosis. In both this study and the US study, ASD diagnoses were confirmed through two-step ASD screening. If either the short or the full version of the scale had been used without follow-up interviews for screen-positive children as in the Norwegian case, the number of FP cases might have increased, and the PPV might have decreased; this possibility needs to be confirmed in future research where short form screening combined with follow-up interviews is prospectively used during community-based health check-ups.

The major limitation of this study was that the data used were not obtained only for the selected 6 items. Since parents might answer differently if they have fewer items to answer, future studies should examine if these items would work similarly to the full version when used on their own prospectively. If these items are in future shown to yield a similar PPV to the full version together with follow-up interviews, then it might be possible to use them as a short form ASD screening instrument at 18 months in primary care settings where time constraints on screening are at a premium, without overloading parents or practitioners should this instrument need to be used repeatedly. The use of this short item set combined with general developmental screening may help to identify probable ASD cases at the young age of 18 months, when used together with follow-up interviews for this less ethnically diverse population. Another important factor to consider is how sociodemographic variables such as maternal education, which we did not collect information on in this study, affect the screening process, since a US study reported that families with lower maternal education also had lower completion rates on the follow-up interviews (Khowaja et al. 2015). Further, in the future, it will also be necessary to examine whether and how the use of this 6-item set containing only sociocommunication behaviors without sensory-related items affects the psychometric properties of the entire M-CHAT(-R)/F screening process according to DSM-5 ASD criteria. While being cognizant of all these issues, the results from this study suggest that this form of integrative approach, where short and quick screening is combined with follow-up interviews, may become one option to heighten community-based developmental surveillance.
